# Myogenic Response to Increasing Concentrations of Ammonia Differs between Mammalian, Avian, and Fish Species: Cell Differentiation and Genetic Study

**DOI:** 10.3390/genes11080840

**Published:** 2020-07-24

**Authors:** Emily Miramontes, Bartosz Kempisty, James Petitte, Srinivasan Dasarathy, Magdalena Kulus, Maria Wieczorkiewicz, Paul Mozdziak

**Affiliations:** 1 Prestage Department of Poultry Science, North Carolina State University, Raleigh, NC 27695, USA; emiramo@ncsu.edu (E.M.); jnppo@ncsu.edu (J.P.); 2 Department of Histology and Embryology, Poznan University of Medical Sciences, 6 Święcickiego St., 60-781 Poznan, Poland; bkempisty@ump.edu.pl; 3 Department of Anatomy, Poznan University of Medical Sciences, 6 Święcickiego St., 60-781 Poznan, Poland; 4 Center of Assisted Reproduction, Department of Obstetrics and Gynecology, Masaryk University, Obilni trh 526/11, 602 00 Brno, Czech Republic; 5 Department of Veterinary Surgery, Institute of Veterinary Medicine, Nicolaus Copernicus University in Torun, Jurija Gagarina 7, 87-100 Torun, Poland; magdalena.kulus@umk.pl; 6 Departments of Gastroenterology, Hepatology and Pathobiology, Cleveland Clinic, Cleveland, 9500 Euclid Avenue, Cleveland, OH 44195, USA; DASARAS@ccf.org; 7 Department of Basic and Preclinical Sciences, Institute of Veterinary Medicine, Nicolaus Copernicus University in Torun, Jurija Gagarina 7, 87-100 Torun, Poland; maria.wieczorkiewicz@umk.pl

**Keywords:** muscle, ammonia, avian, mammals, fish, myostatin

## Abstract

Ammonia is very toxic to the body and has detrimental effects on many different organ systems. Using cultured myoblast cells, we examined ammonia’s effect on myostatin expression, a negative regulator of skeletal muscle growth, and myotube diameters. The objective of this study was to examine how murine, avian, and fish cells respond to increasing levels of ammonia up to 50 mM. The murine myoblast cell line (C2C12), primary chick, and primary tilapia myoblast cells were cultured and then exposed to 10, 25, and 50 mM ammonium acetate, sodium acetate, and an untreated control for 24 h. High levels of ammonia were detrimental to the C2C12 cells, causing increased Myostatin (MSTN) expression and decreased myotube diameters between 10 and 25 mM (*p* < 0.002). Ammonia at 10 mM continued the positive myogenic response in the chick, with lower MSTN expression than the C2C12 cells and larger myotube diameters, but the myotube diameter at 50 mM ammonium acetate was significantly smaller than those at 10 and 25 mM (*p* < 0.001). However, chick myotubes at 50 mM were still significantly larger than the sodium acetate-treated and untreated control (*p* < 0.001). The tilapia cells showed no significant difference in MSTN expression or myotube diameter in response to increasing the concentrations of ammonia. Overall, these results confirm that increasing concentrations of ammonia are detrimental to mammalian skeletal muscle, while chick cells responded positively at lower levels but began to exhibit a negative response at higher levels, as the tilapia experienced no detrimental effects. The differences in ammonia metabolism strategies between fish, avian, and mammalian species could potentially contribute to the differences between species in response to high levels of ammonia. Understanding how ammonia affects skeletal muscle is important for the treatment of muscle wasting observed in liver failure patients.

## 1. Introduction

Myostatin (MSTN) is an important negative regulator of embryonic and postnatal skeletal muscle growth in mammalian, avian, and fish species [1,2]. Myostatin, a member of the transforming growth factor β (TGF-β) super family, affects myogenesis by downregulating myogenic regulatory factor (MRF) expression and inhibits the protein kinase B (Akt)/mammalian target of rapamycin (mTOR)—Akt/mTOR pathway, which then causes a reduction in myoblast proliferation and differentiation [3,4]. Low or absent myostatin expression results in increased levels of hyperplasia and hypertrophy, or the double-muscling phenotype, and high levels of myostatin cause muscle loss [5,6,7,8,9]. The functioning of myostatin in mammalian and avian species has been more extensively studied than in fish species, but the presence of the double-muscling phenotype in myostatin-null fish indicates myostatin has a similar role in skeletal muscle regulation as it does in avian and mammalian species [8,10,11].

Despite similarities between mammalian and fish myostatin, there are also multiple differences that open up possibilities for new roles of myostatin in fish, which remain unknown. Fish have a more extensive expression pattern of myostatin than mammals. Myostatin in fish has been isolated in the heart, eyes, kidney, intestines, and gills, as opposed to the stricter expression to just muscle in mammalian and avian species [2,12,13]. Some fish species have multiple isoforms of myostatin with different patterns of expression, unlike mammalian species with only one copy [13,14,15,16]. The discrepancy between mammalian and fish myostatin makes it more difficult to extrapolate the precise functions of myostatin in fish based solely on studies on mammals.

Previous studies in mammalian and avian species have linked hyperammonemia to changes in myostatin expression. In response to ammonia, mammals exhibit an increase in myostatin expression, and a resultant decrease in skeletal muscle [17,18]. Avian species, however, had a positive myogenic response to hyperammonemia, with a decrease in myostatin expression [18,19]. However, the effects of ammonia on myostatin expression in fish have not been previously considered. Studies have found that high levels of exogenous ammonia have been shown to negatively impact the overall growth of fish, but myostatin expression was not investigated in these studies [20]. 

Fish, mammalian, and avian species all differ in the primary method used to excrete ammonia. Mammalian species are ureotelic and utilize the urea-ornithine cycle (UOC) enzymes primarily found in the liver [21]. Avian species are uricotelic and excrete ammonia as uric acid, primarily depending on glutamine synthetase to detoxify ammonia [22]. Most fish species are ammoniotelic, and excrete ammonia as NH_3_, primarily through the gills [23,24]. However, some fish live in extreme environments and utilize the UOC enzymes to excrete ammonia or use both ammoniotelic and ureotelic excretion strategies [25,26,27]. Since avian and mammalian species differ in the nitrogen excretion strategy and skeletal muscle response to hyperammonemia, it could indicate that fish muscle will also respond differently to high levels of ammonia [18]. The main focus of this study was to examine the effects of increasing levels of ammonia on myogenesis and myostatin expression in mammalian and avian species, and to examine the myogenic response to hyperammonemia in fish. 

## 2. Materials and Methods 

### 2.1. Cells and Cell Culture Media 

The murine myoblast cell line (C2C12) and embryonic day 17 (ED 17) chick breast muscle cells were recovered from frozen storage. The C2C12 and chick cells were plated on 0.1% gelatin-coated 6-well plates and cultured at 37 °C and 5% CO_2_ in proliferation media consisting of 10% fetal bovine serum (Genesee Scientific, San Diego, CA, USA), Dulbecco’s Modified Eagle Medium (DMEM, Gibco, Grand Island, NY, USA), and 1% antibiotic-antimycotic solution (A5955, Sigma-Aldrich, Saint Louis, MO, USA). The cells were grown to approximately 90–100% confluency, with media changed every 48 h.

Tilapia cells were isolated from epaxial skeletal muscle of adult tilapia. The fish was euthanized and then thoroughly cleansed with 70% EtOH and chlorohexidine. A small incision was made with sterile scissors, and 4 pieces of epaxial muscle were excised aseptically. The muscle samples were placed in sterile Hanks Balanced Salt Solution (pH 7.4, HBSS, Sigma-Aldrich, St. Louis, MO, USA). Each muscle tissue sample was mechanically dissociated in the HBSS with sterile scissors and then enzymatically digested with 0.17% trypsin (Sigma-Aldrich) and 0.085% collagenase (Sigma-Aldrich) for 35 min at 37 °C, 5% CO_2_. After incubation, the muscle samples were centrifuged at 18,000 rpm for 5 min. After removing the trypsin-collagenase solution, the samples were washed twice by resuspending the pellet in tilapia proliferation media and centrifuged for 3 min. Proliferation media for the tilapia myoblast cells consisted of 20% FBS (Genesee Scientific), Lebovitz-15 (L-15, Gibco, Grand Island, NY, USA) media, 1% non-essential amino acids (NEAA, Gibco, Grand Island, NY, USA), and 1% antibiotic-antimycotic solution. The cells were resuspended in 3 mL of tilapia proliferation media and passed through an 18-gauge needle, and then through a 70-μm cell strainer (Genesee Scientific). Cell concentration was estimated using a hemocytometer and plated on Matrigel (Corning, Glendale, CA, USA)-coated 6-well plates. The plates were covered with 500 μL of 4 mg/mL Matrigel in DMEM and incubated at 37 °C for 30 min before use. The cells were grown at 27 °C, room air to approximately 90% confluency, with media changed every 24 h. 

C2C12 cells were obtained from ATCC. 217 Perry Pkwy Ste 5, Gaithersburg, MD 20877, United States. All procedures involving live animals were approved by the North Carolina State University Institutional Animal Care and Use Committee. The approval numbers: 19-717-B IACUC approved protocol 10/17/2019.

### 2.2. Differentiation and Treatment Media

After reaching approximately 90% confluency, the media was changed to differentiation media. The C2C12 and chick differentiation media consisted of 10% horse serum (Gibco), DMEM (Gibco), and 1% antibiotic-antimycotic solution (A5955, Sigma-Aldrich). Tilapia differentiation media consisted of 5% FBS (Genesee Scientific), L-15 (Gibco), 1% NEAA (Gibco), and 1% antibiotic-antimycotic solution (A5955, Sigma-Aldrich). The cells were differentiated until they reached 80% differentiation. After reaching 80% differentiation, the 6 well plates were randomly chosen to be treated with treatment media of 10 mM ammonium acetate (AA, Fisher Scientific, Fair Lawn, NJ, USA), 25 mM AA, 50 mM AA, 10 mM sodium acetate (SA, Fisher Scientific, Fair Lawn, NJ, USA), 25 mM SA, 50 mM SA, or a control of untreated differentiation media, with replicates of 8 for each treatment. The sodium acetate control was used in addition to an untreated control to demonstrate that any effect on gene expression is due to the hyperammonemia and not due to the acetate. Differentiation was determined using methods outlined by Davuluri et al. and Kumar et al. [28,29]. The cells in treatment media were incubated for 24 h at 37 °C, 5% CO_2_ for the C2C12 and chick cells, and at 27 °C, room air for the tilapia. After incubation for 24 h, the cells were either fixed and collected for myotube analysis or collected for RNA analysis.

### 2.3. Myotube Diameter

Cells used for myotube diameter analysis were fixed with 70% ethanol for 10 min. The ethanol was removed, and images of the plates were taken with light microscopy at 20× (Leica Microsystems, Buffalo Grove, IL, USA) and a SPOT camera (SPOT Imaging, Sterling Heights, MI, USA). Images from 10 wells per treatment were taken with 10 randomly selected fields of vision per well. For each image taken, one myotube was measured at 3 points equidistant along the myotube, for an average of 100 myotubes measured per treatment for each species. Myotube diameters were measured using ImageJ software (https://imagej.nih.gov/ij/). 

### 2.4. RNA Extraction and Real-Time qPCR

Cells collected for RNA analysis were first washed with 1 mL of HBSS (Sigma-Aldrich). After removing the HBSS, the cells were removed from the bottom of the plate using 0.5 mL of 0.25% trypsin-EDTA solution (Gibco) for approximately 1 min. The trypsin-EDTA was neutralized by differentiation media, the cell suspension spun down, and the differentiation media taken off the cell pellet. The pellet was then resuspended in 500 μL of RNAlater (Thermo Fisher Scientific, Vilinius, Lithuania) and stored at −20 °C until RNA extraction occurred.

Total RNA was extracted from the cell samples following the protocol of the RNAeasy Mini Kit (Qiagen, Hilden, Germany). The RNA was stored at −20 °C in RNase free water. cDNA was then generated following the protocol of the cDNA Reverse Transcriptase Kit (Applied Biosystems, Vilinius, Lithuania) and diluted to a concentration of 1 ng/μL. The diluted cDNA was stored at −20 °C until qPCR was performed. 

Primers for real-time qPCR were designed using primer-BLAST (NCBI). DNA sequencing for tilapia primers was used to verify the qPCR products for each gene (Eton Bioscience, Durham, NC, USA). The sequences of each primer for each gene can be seen in Table 1.

The qPCR was run using 1 μL of 1 ng/μL of cDNA, 10 μL of SYBR Green Mastermix (Applied Biosystems, Warrington, UK), 2 μL of the appropriate primer (10 μm forward and 10 μm reverse primer mix), and 7 μL of water. The qPCR analysis was run on five samples of each cell type and treatment level in triplicate for *n* = 5 replicates. Relative fold changes for each gene were calculated by comparing ammonium acetate to sodium acetate relative to β-Actin using the Pfaffl method [30]. β-Actin was selected as the internal control based on its use in previous studies, with no change in expression in experimental or control treatments, as well as preliminary data (data not shown) demonstrating no change in β-Actin expression in response to AA, SA, or control treatments [19].

### 2.5. Statistical Analysis

Statistical analysis was performed using JMP Pro 15 (SAS Institute Inc., Cary, NC, USA). Myotube diameters were represented as the mean ± SE for ammonium acetate, sodium acetate, and untreated samples. Quantitative real-time PCR was expressed as the mean relative fold change of ammonium acetate-treated samples compared to the sodium acetate-treated samples, relative to the housekeeping gene, β-actin ± SE. All data sets were analyzed by one-way ANOVA and Tukey–Kramer honestly significant difference (HSD) test for multiple comparisons (α = 0.05). 

## 3. Results

### 3.1. Myostatin Response to Ammonium Acetate Differs Between Species

**Figure 1:**
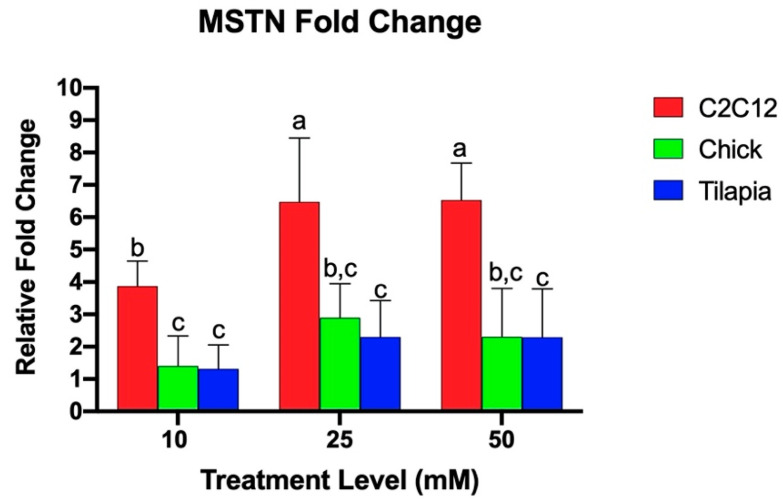
Relative MSTN fold change of gene expression of myotube cells treated with 10, 25, and 50 mM ammonium acetate compared to sodium acetate-treated cells, relative to the housekeeping gene, β-actin, for the tilapia, C2C12, and chick myoblast cells. Letters indicate a significance difference between the relative fold change (*p* < 0.05).

Quantitative real-time PCR was utilized to examine the relative fold change of the gene expression of myogenic markers in response to increasing levels of ammonium acetate for the mouse, chicken, and tilapia. Previous studies found that acetic acid and sodium acetate did not alter myostatin expression in murine myotubes, and any effects on gene expression are due to the hyperammonemia [29]. As seen in Figure 1, C2C12 cells exhibited a higher relative MSTN fold change as compared to the chick and tilapia cells for each increasing treatment (*p* < 0.004). There was no significant difference between tilapia and chick MSTN expressions. The species individually showed different responses to increasing ammonium concentration. C2C12 cells at 25 and 50 mM had significantly higher MSTN expression than C2C12 cells at 10 mM (*p* < 0.002), but the expression between 25 and 50 mM was not significantly different. Tilapia and chick cells each had no significant difference in MSTN expression between increasing treatment levels. 

**Figure 2:**
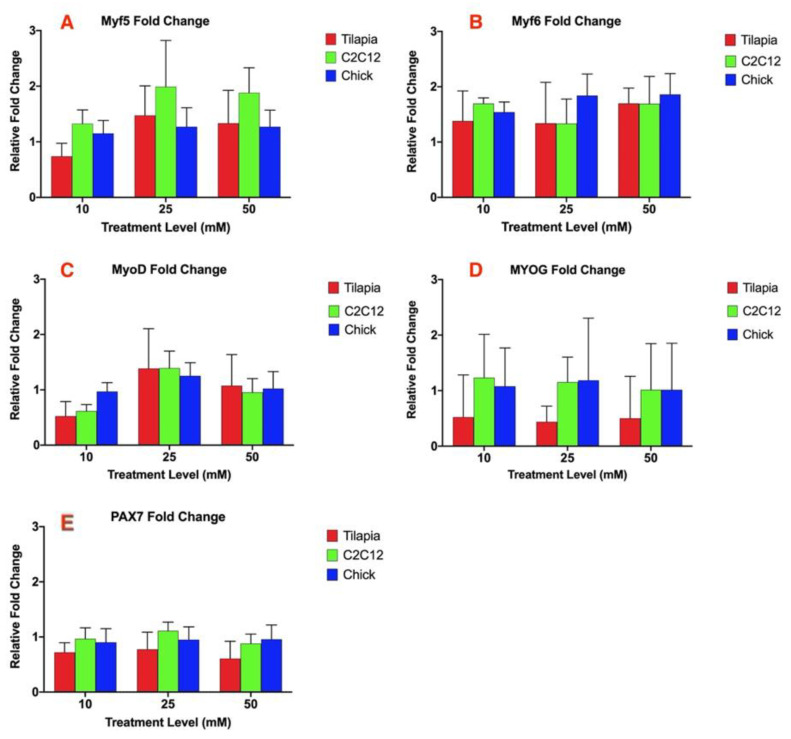
Relative fold change of the gene expression of (**A**) Myf5, (**B**) Myf6, (**C**) MyoD, (**D**) MYOG, and (**E**) PAX7 for myotube cells treated with 10, 25, and 50 mM ammonium acetate compared to sodium acetate-treated cells, relative to the housekeeping gene, β-actin, for the tilapia, C2C12, and chick myoblast cells. There was no significant difference in the expression for each gene between each species or treatment level.

The other myogenic regulatory factors analyzed: myogenic regulator factor 5 (Myf5), myogenic regulatory factor 6 (Myf6), myogenic differentiation factor 1 (MyoD), myogenin (MYOG), and paired box 7 (PAX7), did not result in any significant difference within each species between each treatment at 10, 25, and 50 mM ammonium acetate or between the ammonium acetate and untreated or sodium acetate treatments (Figure 2A–E). There was also no significant difference in the relative fold change of the gene expression between the three species for each MRF analyzed. 

### 3.2. Myotube Diameter Changed in Response to Myostatin Expression for Mammalian and Avian Species

**Figure 3:**
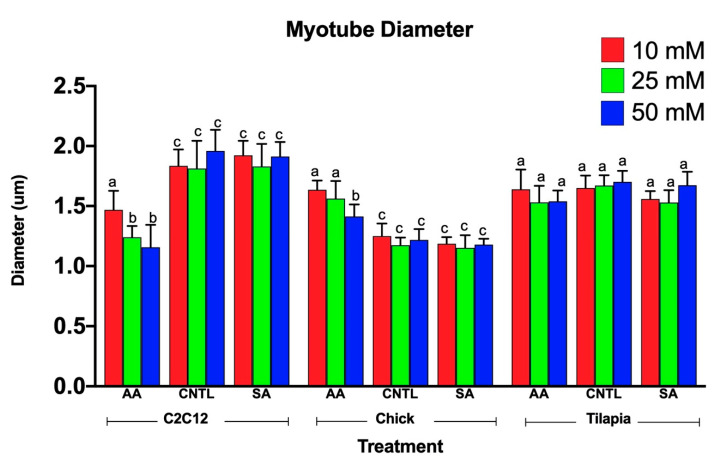
Average myotube diameters for 10, 25, and 50 mM ammonium acetate, sodium acetate, and untreated tilapia, C2C12, and chick myoblast cells. Letters indicate a significant difference within each species (*p* < 0.05).

**Figure 4:**
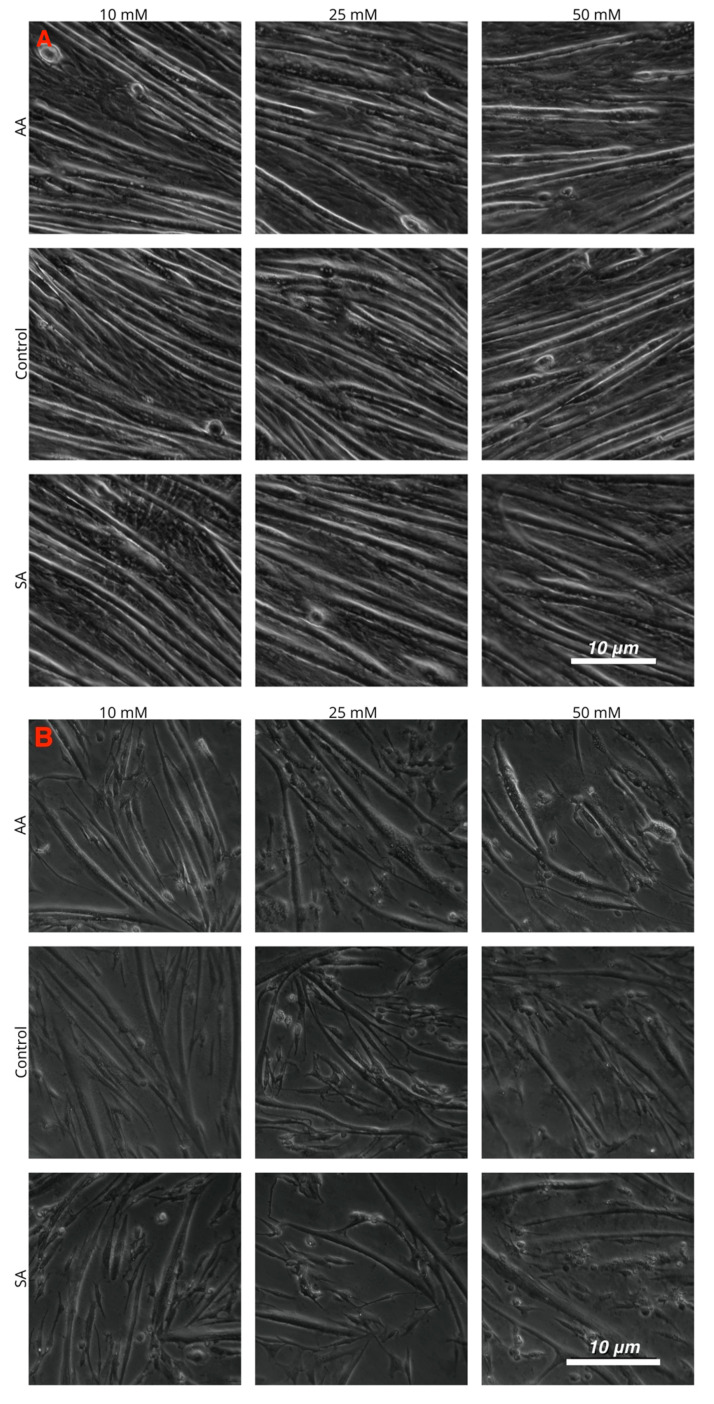

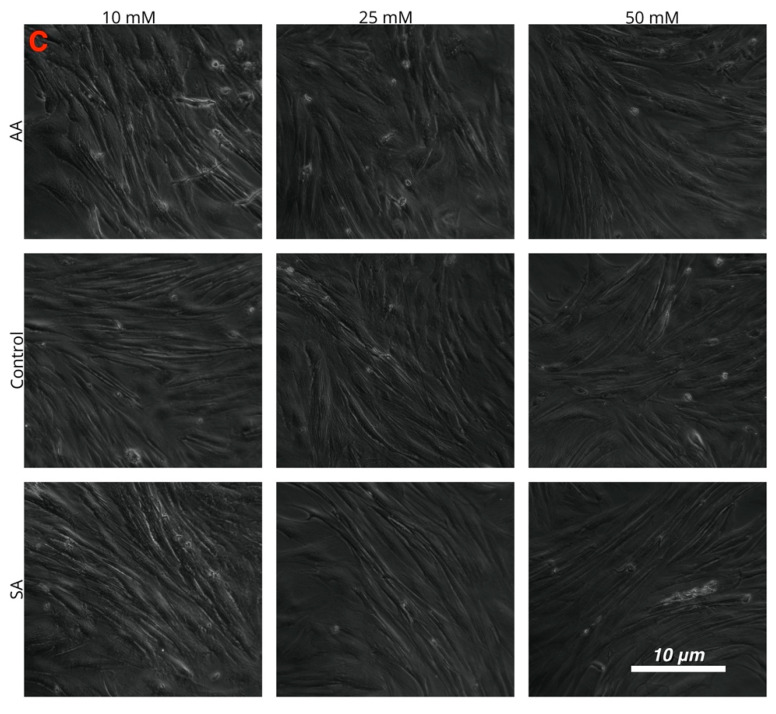
Images of cultured myotubes for (**A**) C2C12, (**B**) chick, and (**C**) tilapia cells after treatment with 10, 25, and 50 mM ammonium acetate, sodium acetate, and proliferation media treatments.

Myotube diameters were measured to examine the myogenic response of the cells to increased ammonium acetate concentrations. As seen in Figure 3, C2C12 myotubes treated with 10, 25, and 50 mM had significantly smaller diameters than those that were untreated and sodium acetate treated (*p* < 0.0001). The 25 and 50 mM C2C12 myotubes were also significantly smaller than the 10 mM-treated myotubes (*p* < 0.008), but there was no significant difference between the 25 and 50 mM treatments. In chick, the ammonium acetate-treated myotubes at 10, 25, and 50 mM for the chick overall had larger diameters as compared to the untreated and sodium acetate-treated myotubes (*p* < 0.001). However, those treated with 50 mM ammonium acetate were significantly smaller than those treated with 10 and 25 mM ammonium acetate (*p* < 0.0001), suggesting that levels above 50 mM may be detrimental to chick myotubes. In the tilapia, myotube diameters were not significantly different between ammonium acetate-treated or untreated and sodium acetate-treated myotubes, or between different treatment levels. Images of the myotubes for each species and treatment are represented in Figure 4A–C.

## 4. Discussion

Previous studies found that at 10 mM of ammonium acetate, C2C12 cells had a significant increase in MSTN expression, as compared to chick myoblast cells at the same level of ammonium [17,18]. The downregulation of MSTN in the avian cells also resulted in significantly larger myotube diameters, while C2C12 cells had significantly smaller myotube diameters [18]. This study examined the effects of titrating the ammonia concentration up to higher levels than previously studied, and how this increase in ammonia affected myogenic gene expression in avian, mammalian, and fish species. While the highest concentration examined in this study was 50 mM, 100 mM of ammonia acetate was also administered but resulted in the death of too many of the C2C12 and avian cells to be able to perform analysis, resulting in the upper level of ammonium used to be 50 mM. The myogenic regulatory factors analyzed did not show any significant difference in gene expression in response to ammonia at 10 mM, as seen in previously published studies [17,18]. The increasing concentrations of ammonia also did not elicit any change in myogenic regulatory factor gene expression in any of the three species.

The results starting at 10 mM were consistent with previous studies for both the C2C12 and the chick cells, with increased MSTN expression for the C2C12 and lower expression in the chick, along with similar myotube diameter results [18]. Increasing the concentration of ammonium acetate to 25 and 50 mM resulted in continued high expression of MSTN in the C2C12 and a further decrease in myotube diameter as compared to the untreated and sodium acetate-treated C2C12 cells. Increased expression of myostatin has been previously linked with a decreasing myotube diameter [18,31,32]. The C2C12 cells continued this same pattern of change between the myostatin expression and myotube diameter. The higher the level of ammonium acetate, the more detrimental ammonia was to skeletal muscle growth. This pattern is also seen in patients with liver failure experiencing muscle wasting due to the high levels of ammonia in the body [33,34].

While the chick had no significant difference in MSTN expression between each treatment, at 25 and 50 mM ammonium acetate, the chick MSTN expression was significantly less than the C2C12 MSTN expression. The myotube diameters for the chick were not significantly different when going from 10 to 25 mM but were significantly smaller at 50 mM. However, the chick myotubes at 50 mM were still significantly larger than untreated and sodium acetate-treated myotubes. The decrease in the chick myotube diameter as the concentration of ammonium increased could be evident of the chick cells starting to feel the toxic effects of ammonia on muscle cells but are able to still regulate the expression of myostatin, as myostatin expression did not increase significantly between 25 and 50 mM ammonium acetate. 

The avian cells at lower levels exhibit a positive myogenic environment with increased myotube diameters in response to ammonium acetate. With increasing levels, there is potentially the beginnings of a negative response to ammonia in the chick myotubes, as the myotubes at the highest concentration on ammonium acetate are smaller than at lower levels of ammonia. However, the myotubes were still larger than those not treated with ammonium acetate. Ammonia could potentially be starting to exert a negative effect on skeletal muscle growth in avian species at higher concentrations outside of myostatin expression, such as osmotic dysregulation seen in astrocytes due to glutamine accumulation [35,36].

The fish showed no significant difference between the three treatments and between the experimental and control samples for the MSTN relative fold change or myotube diameter. Compared to the avian and C2C12 relative fold changes, the tilapia had significantly lower changes in the relative fold change of gene expression than the C2C12 cells but was not significantly different from the avian cells. However, the tilapia cells did not exhibit the positive myogenic response to ammonium that the avian cells exhibited. While the avian cells had an increase in the myotube diameter compared to the untreated and sodium acetate cells, the tilapia did not have any significant difference in the myotube diameters in response to ammonium acetate. Tilapia are specifically able to tolerate a large range of environmental conditions and have been shown to grow well in conditions otherwise toxic to other fish [37,38]. While not every fish has the tolerance observed in the tilapia, the differences in ammonia toxicity between the three species could point to mechanisms in the fish for ammonia detoxification that are different from those in the mammal and avian. 

Fish and avian species have been found to have high levels of glutamine synthetase (GS) in the liver and skeletal muscle, and high levels of ammonia cause an increase in glutamine production [39,40,41,42]. Glutamine is known to inhibit myostatin expression in mammalian and avian cells via decreasing the expression of tumor necrosis factor α (TNF-α) [43]. In avian species, hyperammonemia increased glutamine production in skeletal muscle, and as a result, suppressed myostatin expression as compared to mammals [39]. If fish have high levels of GS activity during high ammonia states, the increased presence of glutamine in skeletal muscle could be the reason myostatin expression and myotube diameter did not change significantly. 

While this study did not focus on glutamine’s effect on the expression of myostatin in the three different species in response to the increasing ammonium levels, future studies should examine glutamine production as a mechanism for mediating ammonia toxicity in skeletal muscle, particularly in fish. It should also be noted the limitation of only examining one species of fish. With the wide variety between different fish species in terms of environment, size, and diet, examining one species does not give a full picture of the effects of ammonia on fish species as a whole. Since many fish species also have multiple isoforms of myostatin, future studies on fish myostatin should also examine the interplay between isoforms. 

These results show context-specific differentiation in the myogenic response to ammonia in different species. Determining the mechanisms of ammonia-mediated skeletal muscle gene responses in different species is important to optimize agriculture and aquaculture production of quality meat. Potentially, for avian species, ammonia could be utilized to a certain level in feed or the environment to increase meat production. It is, however, imperative to evaluate further the effects of ammonia on other organ systems in avian species to ensure it is not toxic to the animal even at levels that may enhance muscle responses. In mammalian systems, ammonia increases myostatin expression, and myostatin-null animals have the potential for greater yields of meat. Therefore, data in mammalian species supports the use of ammonia-lowering strategies to increase lean body and muscle mass with the potential to enhance meat production. Further studies to evaluate differences in gene regulatory responses of ammonia on fish myostatin could reveal novel strategies to regulate environmental or dietary ammonia in order to maximize aquaculture meat production. Increasing recognition of cellular mechanisms on the regulation of gene expression have been studied in mammalian systems focusing on regulatory element interaction within chromosome domains. Species differences in response may be related to differential metabolic and subcellular responses as well as their impact on chromatin interactions and gene expressions. Our studies demonstrate novel species-dependent gene expressions and warrant the use of approaches using chromosome conformational evaluations [44].

## 5. Conclusions

In conclusion, increasing concentrations of ammonia continued to be detrimental to mammalian muscle cells, with smaller myotube diameters and increased myostatin expression. Avian cells showed a decrease in the positive myogenic response to increased levels of ammonia by exhibiting a decrease in myotube diameter when at 50 mM of ammonium acetate. This could indicate the chick cells are approaching a maximum amount of ammonia they can tolerate. Fish cells showed no difference in myogenic response to increased levels of ammonia, indicating the fish cells possess a way of mediating ammonia toxicity that is not present in avian and mammalian species.

## Figures and Tables

**Table 1:** Forward and reverse primers for RT-qPCR.

Species	Primer ^1^	Sequence	Bp Size
Tilapia	MSTN	F 5′-GCTGCGAATGAAGGAAGCTC-3′ R 5′-CGGTGGTCACTTCTTCCGAT-3′	317
Tilapia	MyoD	F 5′-ACGGCATGACGGATTTTAACG-3′ R 5′-CTTGGTAAATCAGGTTGGGGTC-3′	315
Tilapia	Myf5	F 5′-AATGCAAACTACAGCAACGGC-3′ R 5′-GACAGGCGGTCCACGATACT-3′	110
Tilapia	MYOG	F 5′-CAGCAGGGTTTGCTCTACCG-3′ R 5′-CTGAACTGGGCTCGCTTGAC-3′	102
Tilapia	Myf6	F 5′-CCCAAGCGGGTCACGATAAT-3′ R 5′-GCCTTACGTCTATCCGTGGG-3′	160
Tilapia	PAX7	F 5′-GACAGGCGGTCCACGATACT-3′ R 5′-TGCGCCTCTGCTTCCTTTTA-3′	194
Tilapia	β-Actin	F 5′-TGGTGGGTATGGGTCAGAAAG-3′ R 5′-CTGTTGGCTTTGGGGTTCA-3′	217
Murine	MSTN	F 5′-TCACGCTACCACGGAAACAA-3′ R 5′-AGGAGTCTTGACGGGTCTGA-3′	166
Murine	MyoD	F 5′-GCTCTGATGGCATGATGGATT-3′ R 5′-CTATGCTGGACAGGCAGTCG-3′	150
Murine	Myf5	F 5′-AACTATTACAGCCTGCCGGG-3′ R 5′-GCTGGACAAGCAATCCAAGC-3′	198
Murine	MYOG	F 5′-GTGCCCAGTGAATGCAACTC-3′ R 5′-CGAGCAAATGATCTCCTGGGT-3′	94
Murine	Myf6	F 5′-AGAAATTCTTGAGGGTGCGG-3′ R 5′-GCCCCTGGAATGATCCGAAA-3′	76
Murine	PAX7	F 5′-AGTTCGATTAGCCGAGTGCT-3′ R 5′-CATCCAGACGGTTCCCTTTGT-3′	142
Murine	β-Actin	F 5′-AGATCAAGATCATTGCTCCTCC-3′ R 5′-AGCTCAGTAACAGTCCGCCTA-3′	170
Avian	MSTN	F 5′-CGGAGAATGCGAATTTGTGTTTC-3′ R 5′-GGGACATCTTGGTGGGTGTG-3′	110
Avian	MyoD	F 5′-CGCAGGAGAAACAGCTACGA-3′ R 5′-ATGCTTGAGAGGCAGTCGAG-3′	104
Avian	Myf5	F 5′-TGAGGGAACAGGTGGAGAACT-3′ R 5′-ACTCTGCTCCGTCGCGTA-3′	185
Avian	MYOG	F 5′-CAGCCTCAACCAGCAGGAG-3′ R 5′-ACTGCTCAGGAGGTGATCTG-3′	166
Avian	Myf6	F 5′-AGGCTGGATCAGCAGGACAAAA-3′ R 5′-CGCGGGAATGGTCGGAAG-3′	139
Avian	PAX7	F 5′-GAAGGCCTTTGAGAGGACCC-3′ R 5′-GGTTGAATGCTGCGAGTTGG-3′	158
Avian	β-Actin	F 5′-GTCCACCTTCCAGCAGATGT-3′ R 5′-TAAAGCCATGCCAATCTCG-3′	168

^1^ Gene names are defined as myogenic differentiation factor 1 (MyoD), myogenic regulator factor 5 (Myf5), myogenin (MYOG), myogenic regulatory factor 6 (Myf6), and paired box 7 (PAX7).
